# Effect of Atmospheric Temperature Variations on Glycemic Patterns of Patients with Type 1 Diabetes: Analysis as a Function of Different Therapeutic Treatments

**DOI:** 10.3390/ijerph22121850

**Published:** 2025-12-11

**Authors:** Alessandra Mascitelli, Stefano Tumini, Piero Chiacchiaretta, Eleonora Aruffo, Lorenza Sacrini, Maria Alessandra Saltarelli, Piero Di Carlo

**Affiliations:** 1 Department of Advanced Technologies in Medicine & Dentistry, Center for Advanced Studies and Technology—CAST, University “G. d’Annunzio” of Chieti-Pescara, Via dei Vestini, 31, 66100 Chieti, Italy; alessandra.mascitelli@unich.it (A.M.);; 2 CNR-ISAC, Institute of Atmospheric Sciences and Climate (ISAC), National Research Council (CNR), Via del Fosso del Cavaliere 100, 00133 Rome, Italy; 3 Department of Maternal and Child Health, UOSD Regional Center of Pediatric Diabetology, “SS. Annunziata” Hospital, 66100 Chieti, Italy; stefano.tumini@asl2abruzzo.it (S.T.);; 4 Department of Sciences, Center for Advanced Studies and Technology—CAST, University “G. d’Annunzio” of Chieti-Pescara, Via dei Vestini, 31, 66100 Chieti, Italy; eleonora.aruffo@unich.it

**Keywords:** atmospheric temperature, type 1 diabetes, climate change, glucose pattern, insulin pump

## Abstract

An overview of seasonal variations in glycaemic patterns in children and young adults with type 1 diabetes has been addressed in a previous work, which paved the way for an in-depth study involving not only traditional Multiple Dose Injection (MDI) therapy, but also a comparative analysis with the use of Advanced Hybrid Closed-Loop (AHCL) insulin pumps. The widespread use of Flash Glucose Monitoring (FGM) and Continuous Glucose Monitoring (CGM) systems, as well as dedicated platforms for synchronizing and storing CGM reports, has facilitated an efficient approach to analyzing glycaemic patterns. The effect of environmental parameters on glycemic trends undoubtedly has a clinical relevance, which however can be appropriately managed by knowing the responses in patients treated with different therapeutic approaches. In this sense, it is possible to evaluate how the glycemic trend in diabetic patients, in relation to external temperatures, responds differently to therapies. In this work, the response, in terms of glucose level, in diabetic patients was analyzed, according to the different therapeutic approaches and in relation to variations in external temperature. For the same period of the previous work (one year: Autumn 2022–Summer 2023), seasonal variations in CGM metrics (i.e., Time In Range—TIR, Time Above Range—TAR, Time Below Range—TBR and Coefficient of Variation—CV) were analyzed. The results show a better metabolic control, linked to the effect of the algorithm on the trend of glycaemia. However, the analysis focused on the heatwave of July 2023 highlights the role of extreme temperatures as a stress factor in the insulin pumps performance. A further focus was carried out on the comparison of glycaemic patterns during the school and non-school period for all patients until 21 years old. Results suggest that during the school period, glycaemic patterns, in patients treated with MDI, show a greater onset of hyperglycaemia. From all that has emerged, it appears clear that structured education on diabetes self-management for patients and their families is fundamental and must take into account multiple factors (type of therapy, daily activities, atmospheric temperature) in order to keep their effects under control.

## 1. Introduction

In recent years, the treatment of type 1 diabetes T1D has seen the introduction of innovative technologies that have improved metabolic control, with benefits in terms of acute complications (hypoglycaemia, ketoacidosis DKA) and chronic complications (micro and macroangiopathy), as well as improvement in quality of life [1,2]. This study extends previous work [3,4] by including patients treated with both MDI and AHCL systems, analysing a full year of CGM data, and integrating seasonal, heatwave and behavioural (school vs. non-school) contexts. Unlike earlier studies focusing only on seasonal variation in paediatric MDI users, our work allows direct comparison between treatment modalities and across environmental and behavioural conditions. However, childhood and adolescence in particular remain critical stages in the natural history of the disease. During adolescence, glycaemic control worsens [5], partly because psychological support is often insufficient or absent, even in the wealthiest healthcare systems. Psychosocial and socioeconomic factors certainly play an important role. In addition to factors such as fear of hypoglycaemia [4], health education levels are often affected by a lack of professional resources for patient education [6]. Furthermore, with conventional systems, monitoring during school hours and fear of hypoglycaemia have often contributed to the acceptance of excessively high blood glucose levels. Frequently, the use of technology is the result of a self-taught approach, partly due to a lack of professionals who are up to date with training patients and families. In fact, advanced technologies (closed-loop, HCL, etc.) are evolving so rapidly that resources for professional development are often insufficient. The sheer volume of data available on shared platforms also requires self-management skills and IT literacy that are not within everyone’s reach, and eHealth illiteracy has a particularly negative impact on the role of parents [7]. In disadvantaged and/or immigrant families with cultural integration problems, already compromised health literacy can increase the gap in terms of adequate and optimal self-management knowledge, especially in younger children. Optimal use of technology requires not only adequate individual and family motivational resources, but also knowledge of aspects relating to nutrition, diet, sports activities and specific knowledge relating to the management of insulin pumps, knowledge of how to manage algorithms even in particular situations [8], and the management of the infusion site, which is often problematic in paediatric patients [9,10]. Recent studies show that climate change and heat waves also have a significant impact on glycaemic control [11,12,13,14,15]. Seasonal variations in glycaemic control and acute complications (hypoglycaemia and DKA) have been the subject of scientific evaluation, and studies in the literature have demonstrated increased morbidity and cardiovascular risk, especially in adults [11,12,16,17,18,19,20]. A higher incidence of hypoglycaemia and ketoacidosis has been demonstrated in adults with type 2 diabetes (T2DM) [14,21]. The prevalence of hypoglycaemia has been correlated in adults with type 1 diabetes with an 8% increase in hospitalisation due to a 5 °C increase in daily temperature, with more frequent hypoglycaemia in summer [14,21]. There are few paediatric studies [16,22]. The results of our previous study [3] show that, during heat waves, there are pronounced deviations in blood glucose levels, indicating significant effects of extreme temperatures on glucose levels. The results obtained have highlighted the need to integrate meteorological parameters into diabetes management and clinical trial designs [23]. Furthermore, it is clear that structured education on diabetes self-management for patients and their families must include adequate information on the effects of extreme temperature variations, especially on the risk of hypoglycaemia [24]. Furthermore, excessive therapeutic inertia associated with FoH (Fear of Hypoglycaemia) in adjusting insulin doses during rapid climatic changes can result in worsening metabolic control [4,25]. This study aims to assess whether the advantages of AHCL systems on metabolic control [1] are confirmed in relation to seasonality, during heat waves and during the school period, which is generally associated with a worsening of glycaemic control compared to holidays [26,27].

## 2. Materials and Methods

### 2.1. Research Design

In order to make the research as representative as possible, two groups of patients were involved in the analysis: a MDI group of 138 subjects with type 1 diabetes (aged up to 21 years) and an AHCL group of 81 patients (of whom only 47% were under 21 years). This is a retrospective observational cohort study including the entire eligible population, therefore no a priori sample size calculation was performed; all patients are followed at the UOSD Regional Paediatric Diabetes Service Hospital ‘SS. Annunziata’ Hospital, MDI group use the Freestyle 2-3^®^ system and have a high adherence to continuous glycaemic monitoring (>70%). The data used are synchronised on the cloud-based management system (Freestyle^®^ LibreView system) and shared with the diabetes care team. The AHCL Group used Advanced Hybrid Closed Loop (AHCL) systems, including: XX MiniMed™ 80G with Guardian™ 4; XX Tandem t:slim X2—Control-IQ System with Dexcom G6; XX YpsoPump—mylife CamAPS FX with Dexcom G6. To achieve the desired glycemic targets, AHCL pumps use three main systems, distinguished by the type of algorithm implemented: Proportional-Integral-Derivative (PID) control, Model Predictive Control (MPC), and Fuzzy Logic. Additionally, these algorithms are equipped with predictive safety modules designed to limit insulin delivery and prevent hypoglycemia episodes [28]. The inclusion criteria considered relate to the diagnosis of T1D (Type 1 Diabetes) confirmed by the positivity of at least one of the antibodies against islet cells (ICA), insulin (IAA), glutamate dehydroxylase (GADA), islet antigen 2 (IA2A) and zinc transporter protein 8 (ZnT8A). Exclusion criteria include patients with diabetes duration < 1 year, more than one episode of DKA in the last year, uncontrolled celiac disease or hypothyroidism, oncohaematological diseases, severe, psychiatric disorders or systemic steroid therapy during the study year. The glycaemic profiles were assessed in relation to the atmospheric temperature (provided by the Regional Hydrographic Office.) relative to the patients’ area of residence, thus considering seasonal and diurnal trends. The evaluation was conducted considering different therapeutic approaches (MDI and AHCL) in order to assess their interactions with the atmospheric factors considered (temperature trends in the study area and extreme phenomena of the same parameter: heatwave). A further point analysed was the impact of the weekday and holiday period on glycaemic trends, with particular reference to paediatric patients. In order to conduct an accurate statistical analysis, the study was conducted at different scales: annual (Autumn 2022–Summer 2023), seasonal, diurnal and single-event (heatwave). Seasonal variations in the CGM metrics, means, standard deviations, medians, maximum values, RMSE, MAD, scatter plots, correlation coefficients and box plots were analysed.

### 2.2. Medical Data

On 1 September 2024, the part of the dataset referred to patients treated by insulin pump was provided to the research group, in a pseudonymized format and for research purposes only, in consistency with what was performed with the part of the dataset described in a previous publication [3]. The study focuses on the time period from 20 September 2022 to 21 September 2023, for which both part of dataset are available. Quality check has been carried out on the whole dataset, showing a consistent and mostly complete dataset (>70% for all time spans considered). Demographical and clinical characteristics of participant included in the two studied clusters are compared in Table 1. Being the prevalence of diabetes in preschool-aged children (0–6 years) relatively low, the number of potential pump users in this age group is naturally limited. In addition, only in recent years the introduction of AHCL systems increased the acceptance and prescription of insulin pumps in children aged 6–11 years. Furthermore, several pump models are not approved for use in children under 6 years of age [28]. However, in order to make the analyses more reliable and to address the age imbalance between groups, the analysis on differences between working days and public holidays was performed focusing on the subgroup cluster restricted to participants aged ≤21 years.

### 2.3. Meteorological Data

As in our earlier work [3], we relied on raw meteorological temperature data provided by the Regional Hydrographic Office to ensure methodological consistency. These data consist of instrumental temperature measurements collected by all available monitoring stations between 1 January 2022 and 31 December 2023. The dataset—directly acquired from the data-logging systems of the 102 stations—includes the date, solar time, and temperature values averaged over 15-min intervals. For this study as well, a thorough quality-control procedure was applied to the temperature records. This process involved identifying and addressing potential issues such as missing values, anomalous spikes, or other irregularities to improve the overall reliability of the dataset. The analyses presented here make use of data from the full network of stations, restricted to the period spanning from 20 September 2022 to 21 September 2023.

## 3. Results

### 3.1. Annual Analysis

**Figure 1:**
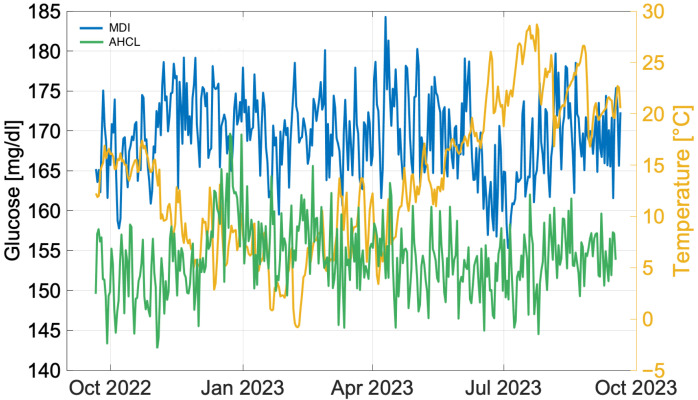
Daily mean glucose levels (both MDI and AHCL) and atmospheric temperature variations (Autumn 2022–Summer 2023). The graph combines medical data and temperature data to highlight trends and correlations over the specified period.

A first analysis was performed on the annual pattern in mean daily glucose, in occurrence of MDI and insulin pump use, and atmospheric temperature. Temperature was analysed as the sole environmental exposure because it was the only meteorological parameter with complete hourly coverage across all 102 stations, whereas humidity, wind speed and precipitation had substantial missing data and collinearity. The daily glucose averages of the 138 patients treated with MDI and the daily glucose averages of the 81 patients treated with insulin pumps were computed for the period from 20 September 2022 to 21 September 2023. In this case, whole cluser treated with AHCL was considered, being the purpose to evaluate the long-term effect of temperature on the type of treatment adopted. The resulted patterns are shown, in comparison with also temperature trend, in Figure 1, showing significantly lower blood glucose values when using insulin pumps, compared to MDI.

**Figure 2:**
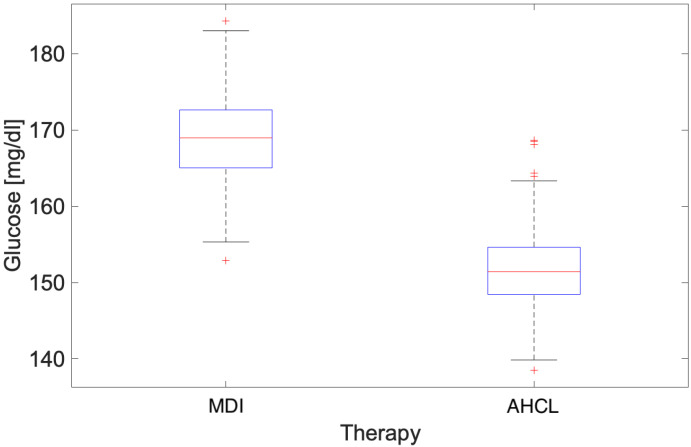
Glucose level box-plot of both MDI and AHCL referred to the period Autumn 2022–Summer 2023. The central red line indicates the median; the lower and upper edges of the box represent the 25th and 75th percentiles, respectively; the whiskers extend to the most extreme non-outlier values; outliers are shown as red crosses (+).

A box-plot comparing the glycaemic values referring to the two treatments (MDI and AHCL) is given as shown in Figure 2. As can be seen from the plot, the median of glycaemic values in patients treated with insulin pumps is clearly lower than that obtained from values recorded in patients treated with MDI. Furthermore, the smaller distance between the 25 and 75 percentile segments shows the smaller dispersion of values from the median. At this point, an analysis of glycaemic patterns during different seasons was carried out, in order to better understand the potential effect of atmospheric temperature also in relation to the two different medical treatments. Also in this case, the parameters for clinical practice [29] defined by International Consensus were employed:

- Number of days the sensor was worn (recommended ≥14 days)
- CGM utilization rate (recommended >70%)
- Average blood glucose
- Estimated glycated haemoglobin
- Glycaemic variability (target CV ≤ 36%)
- TAR (>250 mg/dL) [Level 2]: <5%
- TAR (181–250 mg/dL) [Level 1]: <25%
- TIR (70–180 mg/dL): >70%
- TBR (54–69 mg/dL) [Level 1]: <4%
- TBR (<54 mg/dL) [Level 2]: <1%

Considering these definitions, an initial filtering was performed, using the first two conditions, before assessing the glycaemic variability for each season; at this point, another filtering based on it was applied, eliminating all patients characterised by a CV > 36% (Table 2).

Although this last operation (shown in Table 2 for the different seasons) affected the sample size, it made it possible to exclude potential outliers. Moreover, the comparison between the treatments highlights how the use of insulin pump increases the percentage of CV target patients. The chi-square test performed on the data, grouped by season and type of therapeutic treatment, confirmed that there is a statistically significant association between CV and type of treatment (*p* < 0.01). As for the previous study [3], seasonal differences in glycaemic patterns and frequency of hypoglycaemic and hyperglycaemic episodes were analysed using the standard International Consensus metrics (TIR, TAR level 1 and level 2 and TBR level 1 and level 2) (Table 3).

TIR and TAR results showed a percentage of time spent in target, in every seasons, higher in correspondence of the insulin pump use than MDI, in line with the result shown shortly before. Conversely, TBR results shows a percentage of time in target lower in almost every seasons, in occurrence of insulin pump use, with the only exception of first level during the summer season. This is coherent with the results of previous study [3], in wich the frequency of hypoglycaemic events was almost negligible. This fact is also confirmed by the yearly pattern analysis as shown in Figure 1.

### 3.2. Heatwave Analysis

**Figure 3:**
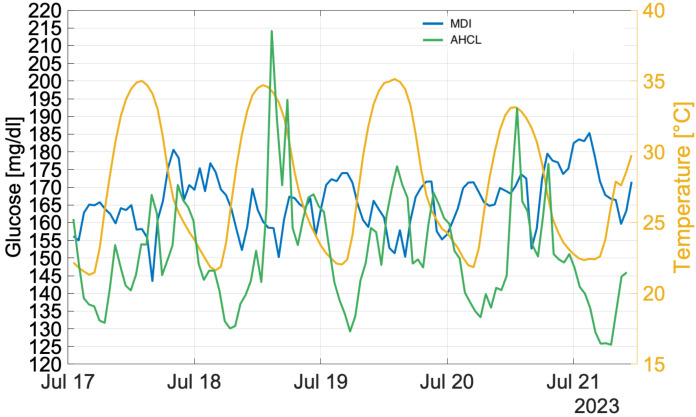
Glucose levels (both MDI and AHCL) and atmospheric temperature variations (17 July–21 July 2023).

**Figure 4:**
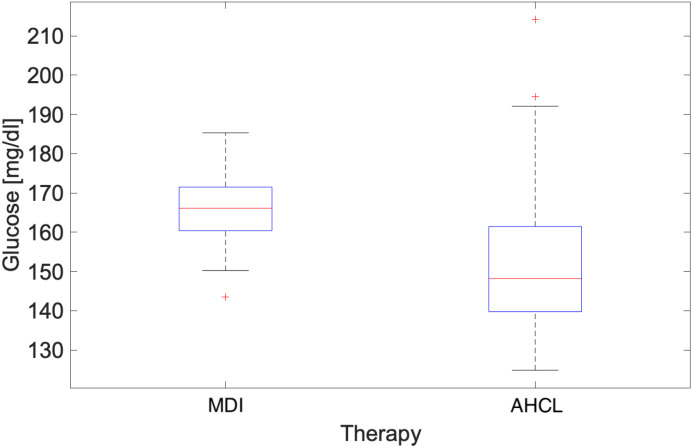
Box-plot glucose levels (mg/dL) for both MDI and AHCL referred to the heatwave period (17 July–21 July 2023). The central red line indicates the median; the lower and upper edges of the box represent the 25th and 75th percentiles, respectively; the whiskers extend to the most extreme non-outlier values; outliers are shown as red crosses (+).

In this section, a comparative analysis of the two treatments response in occurrence of the July 2023 heatwave [3,30,31,32,33] is given. Also in this study, a focus was performed over the period 17–21 July 2023 as shown in Figure 3, comparing atmospheric temperature and glucose trend timeseries [3]. And also in this case, whole cluster treated with AHCL was considered with the aim to understand the effect of extreme temperature on the type of treatment adopted. During this period, the averaged difference between hourly values and the monthly mean temperature was +3 °C [3].The distribution of glucose values during the heatwave for both MDI and AHCL treatments is shown in Figure 4.

A statistical analysis of the differences between MDI and AHCL in the two periods (one year and heatwave 2023) has been carried out (Table 4).

Even a first glance at the statistical indices, in particular the difference between the RMSE values, shows how the variability of our data set changes during the extreme event. The Median Absolute Deviation (MAD), which is the most robust measure of variability for outliers (it is calculated by taking the median of the absolute deviations of each data point from the median of the data set), shows how spread out the data is from the median. As can be seen from the Table 4, the variability of the differences between the data for the two treatments increases significantly (factor 3) during the heat wave. This suggests a different behaviour, compared to the standard, of the insulin pumps during extreme temperature events.

### 3.3. Working Days and Public Holidays Analysis

**Figure 5:**
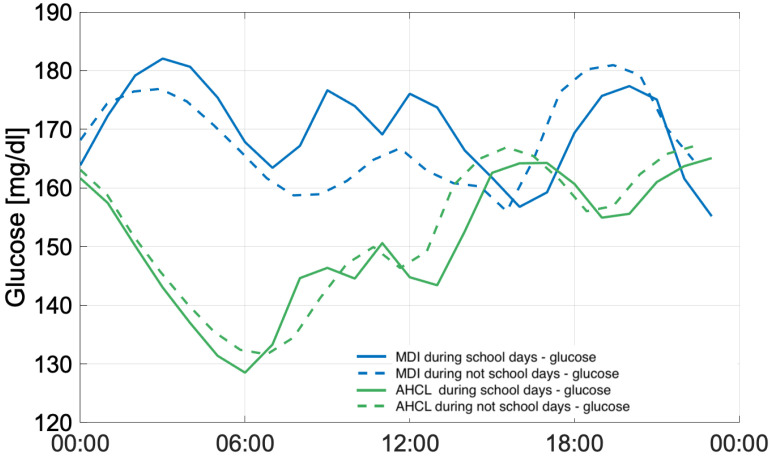
Glucose levels (mg/dL) patterns for both MDI and AHCL referred to the school period and not school period.

An additional analysis referred to the comparison between patterns during working days and public holidays periods was performed. In this case, the cluster of patients treated by insulin pump was employed as potential reference for AHCL wanting to assess the effect of time spent at school out of the families’ control, but considering, in this frame, that only 47% of the sample is under 21 years of age. Therefore, in this case only patients ≤ 21 belonging to the cluster treated with AHCL were considered. Despite the difference in terms of numerosity, the procedure was still considered valid because, as the data show, the greatest effect occurs where there is actual human intervention (MDI). In this regard, the following procedure was therefore adopted. Following a separation of school (working days) and non-school (public holidays) days, the plot in Figure 5 was derived, comparing the two diurnal trends in the case of MDI for the two different periods (blue—school days, dashed blue—non-school days) and pumps for the two different periods (green—school days, dashed green—non-school days) as shown in Figure 5.

**Figure 6:**
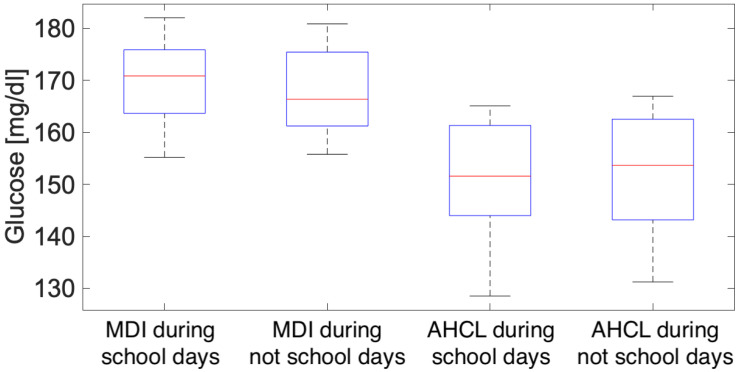
Box-plot glucose levels (mg/dL) for both MDI and AHCL referred to the school period and not school period. The central red line indicates the median; the lower and upper edges of the box represent the 25th and 75th percentiles, respectively; the whiskers extend to the most extreme non-outlier values.

With reference to these trends, a box-plot comparing the blood glucose values for the two treatments and the two school/non-school periods was produced. The impact of the school days on the blood glucose pattern in the case of the MDI (higher median, bigger extremes) is evident from this picture, whereas the blood glucose pattern with the AHCL seems to be minimally affected as shown in Figure 6.

A SID (Italian Society of Diabetology) metrics computation (Table 5) was conducted on the two patient groups in order to compare the four scenarios as shown in Figure 6.

The values in the Table 5 show a better average performance when using insulin pumps. Even more striking, however, is the general tendency to have a lower percentage of patients with time on target for hyperglycaemic events (TAR level 1 and level 2) when using MDI during the school term (67% compared with values above 75% and 78% compared with values above 93%—see Table 5). A comparative statistical analysis table on the differences between the two patterns in MDI (school days-not school days) and AHCL (school days-not school days) is shown (Table 6).

In this case it is clear how the variability of the differences between the data for the two periods increases significantly (factor 2) when MDI is used. This behaviour suggests that, in the case of the use of MDI, the school period has its own impact on the onset of hyperglycaemic phenomena.

## 4. Discussion

Unlike previous technologies that allowed for intermittent blood glucose monitoring (SMBG—Self Monitoring of Blood Glucose) [34], the availability of wearable sensors and data synchronisation systems has enabled long-term analysis of blood glucose trends, thanks in part to the introduction of shared metric systems for report analysis, such as AGP (Ambulatory Glucose Profile), and the definition of blood glucose targets [29,35]. Many available studies have only considered variations in HbA1c levels in school-aged children with type 1 diabetes, demonstrating seasonal variability in HbA1c that could influence the results of clinical studies, as well as the diagnosis of diabetes based solely on HbA1c [23]. Many of the metabolic parameters detectable with CGM were not detectable with HbA1c alone, even though the need to implement knowledge of the disturbances induced by seasonal variability and heat waves in therapeutic education programs had already been emphasized [23]. Studies evaluating seasonal variations in terms of metabolic control and acute events (hypoglycemia and hyperglycemia) remain scarce in type 1 and type 2 diabetes [11,12,13,14,15], particularly in children [22]. However, the available data show a correlation between metabolic control and seasonal temperature variations and heat waves, as well as episodes of hypoglycemia and hyperglycemia. Lanzinger points out that in children, rising temperatures correlate with a reduction in second-level TAR [22]. In our previous work on subjects with MDI, we highlighted how seasonal glycemic variations are responsible for significant changes in metabolic control, with an anti-correlation between temperatures and average blood glucose levels throughout the year, with a higher prevalence of hypoglycemic episodes, especially during the transition from cold to warm seasons, also due to increased physical activity [3,36,37]. Furthermore, we hypothesized that the use of fixed insulin regimens and excessive latency in adjusting insulin doses to seasonal variations and heat waves exposed patients to a risk of deterioration in glycemic control, including a higher incidence of hypoglycemia [3,15,22,25]. In this study, we focused our attention on modern AHCL technologies, and our results demonstrate better metabolic control compared to MDI-CGM regardless of the season. Furthermore, we also highlight a reduction in glycemic variability throughout the year. This finding appears relevant in light of numerous studies highlighting the role of glycemic variability in the increased risk of hypoglycemia and the development of chronic microangiopathic complications [38]. AHCL systems increase the percentage of patients who achieve their desired glycemic targets, with the resulting benefits in terms of the risk of developing complications. As in the previous study [3], seasonal glycemic differences and the frequency of hypoglycemic and hyperglycemic episodes were analyzed using standard parameters (TIR, TAR level 1 and level 2, and TBR level 1 and level 2) [29]. The population with insulin pumps had a higher percentage of individuals with TIR and TAR values within the recommended targets than the population on MDI. In contrast, TBR results show a lower percentage of time in target in almost all seasons when using AHCL systems, with the sole exception of first-level TBR during the summer season. This is consistent with the results of our previous study [3], in which the frequency of hypoglycemic events was, however, almost negligible. This data is also confirmed by the analysis of the annual trend. Furthermore, the anticorrelation of glycemic values already highlighted in the literature is confirmed [3,22]. These results indicate, however, the need to adequately educate families on the importance of promptly and actively managing the settings of AHCL systems, avoiding a wait-and-see attitude even during heat waves. It is therefore important to emphasize the need to adjust parameters such as glycemic targets, ISF (Insulin Sensitivity Factor), ICR (Insulin Carbohydrate Ratio), Insulin Duration (ID), and, where necessary, update the subject’s weight [25]. In addition, particular attention should be paid to educating patients on the correct use of functions that allow for acute modulation of algorithm aggressiveness (booster functions, physical activity, etc.) [39]. These results indicate the need for further educational measures on the use of closed-loop insulin delivery. In a recent study, AHCL systems did not show a reduction in cases of hypoglycemic coma, but a significant reduction in the rate of severe hypoglycemia with an improvement in average blood glucose levels and an increased risk of ketoacidosis. This study [40] also reiterates the need for further educational measures on the use of closed-loop insulin delivery, including for the management of seasonal climate variations and heat waves. In relation to glycemic variability, AHCL systems show clear superiority over MDI-CGM, with percentages of subjects at target (CV < 36%) fluctuating between 57–62% and 30–31% respectively over the four seasons (Table 2). Reduced glycemic variability has also recently been associated with the use of AHCL systems [40]. Outdoor temperature was selected as the primary environmental variable because it was the only meteorological parameter consistently available for all monitoring stations (102) throughout the study period. Other factors such as humidity, wind speed and precipitation were excluded due to incomplete coverage and strong collinearity with temperature. This represents a limitation, as does the lack of complete homogeneity between the two samples, the difference between which is linked to the therapeutic treatments that can be applied for different age groups. To sum up, this study has clear limitations. First, the retrospective design does not allow causal inference. Second, the comparative groups differed in age and baseline characteristics; although age-restricted and adjusted analyses were performed, residual confounding may persist. Third, only air temperature was analysed, due to incomplete availability of other meteorological parameters. Finally, the study was conducted in a single region, limiting generalisability.

### 4.1. Annual Analysis

The comparison between treatments shows that the use of the AHCL insulin pump Systems increases the percentage of patients who achieve glycemic targets. As in the previous study [3], seasonal differences in glycemic patterns and the frequency of hypoglycemic and hyperglycemic episodes were analyzed using standard parameters (TIR, TAR level 1 and level 2, and TBR level 1 and level 2) (Table 3). This is consistent with the results of a previous study [3], in which the frequency of hypoglycemic events was still almost negligible and within a range that correlates in many families and patients with the motivated and effective pursuit of control very close to the targets considered optimal. This data is also confirmed by the analysis of the annual trend as shown in Figure 1; Table 2). In particular, for CV, a greater number of subjects are observed to be on target compared to MDI in all seasons, with only 30% of subjects > 36% CV in the MDI group compared to 60% in the AHCL group. There is evidence that better glycemic control associated with reduced glycemic variability and a reduced number of severe hypoglycemic episodes may contribute to improving quality of life through reduced fear of hypoglycemia and a reduction in the daily burden that technology can offer. Many recent studies seem to confirm that AHCL systems in particular are able to guarantee better results in this regard [1]. Furthermore, the use of these systems can significantly reduce the time spent on diabetes care, especially in younger patients [41,42]. It has been shown that pediatric age, particularly in the preschool period, is associated with increased use of remote management software, although less than the time required for therapy management with manual systems. This could indicate a need for greater attention in this age group due to greater glycemic variability, a reduction in the workload of family members for control-related activities (insulin preparation, blood glucose measurement, etc.) [43] or the propensity of younger generations of parents to use technology, but also the perceived ability of these systems to improve outcomes in terms of both metabolic control and quality of life.

### 4.2. Heatwave Analysis

Heatwave analysis shows that in MDI and AHCL patients, glycemic variability expressed as Root Mean Squared Error (RMSE) shows distinct trends. The Median Absolute Deviation (MAD), which is the most robust measure of variability for outliers (calculated by taking the median of the absolute deviations of each data point from the median of the data set), shows how widely the data are distributed from the median. Furthermore, the variability of the differences between the data for the two treatments increases significantly (Table 4) during the heatwave. This suggests a more effective and protective functioning of the AHCL group compared to MDI, with a favorable difference for all trend and dispersion indicators compared to MDI. This data may have important clinical significance in light of reports of more frequent episodes of metabolic decompensation and severe hypoglycemia related to high temperatures [11,12,13,14,15].

### 4.3. Working Days and Public Holidays Analysis

The analysis comparing the models during working days and holidays shows that the group of patients treated with an insulin pump in order to assess the effect of time spent at school outside the control of their families, although it should be noted that only 47% of the sample is under 21 years of age. However, it was considered a valid procedure because, as the data show, the greatest effect occurs where there is actual human intervention (MDI). In this regard, the following procedure was therefore adopted. Following a separation between school days (working days) and non-school days (holidays), the graph in Figure 5 was obtained, which compares the two daily trends in the case of MDI therapy for the two different periods (blue—school days, dashed blue—non-school days) and AHCL for the two different periods (green—school days, dashed green—non-school days) as shown in Figure 5. These data highlight that glycemic control is always better in the AHCL group during the school period than in the MDI group. This is the first observation to show the superiority of AHCL during the school period. This observation is important and indicates the potential contribution of AHCL systems to improving glycemic control during school hours. Hyperglycemia during school hours was common in patients (aged 4–19 years) who used CGM [44]. In a cohort of children (aged 7–12 years), TIR was higher and TAR lower on weekends [45]. In another study, patients using intermittently scanned Continuous Glucose Monitoring (isCGM) showed poorer control during holidays [46]. In this sense, it is believed that AHCL therapy can actively contribute to improving glycemic control during school hours, considering that children spend about a quarter of their day at school and that the ISPAD-IDF guidelines clearly indicate the need to improve metabolic control during school hours. Furthermore, it should be emphasized that, especially in adolescents, the discretion of AHCL systems improves compliance with therapy, both with the possibility of active intervention and with automatic interventions by algorithms. It should not be overlooked that the ability of primary caregivers to stay informed about glycemic trends, including glycemic trends, hypoglycemia, pump automatisms, and active insulin, allows them to provide real-time support and guidance that reassures school staff, especially if they are not trained and experienced in managing children with diabetes. The advantage offered by AHCL systems persists even during school hours, which are often subject to unexpected reductions in insulin doses for those using MDI in order to reduce the risk of hypoglycemia, particularly in cases where there is no cooperation from the school. It is important to note, however, that the MDI population has a lower percentage of patients with TAR values of I and II levels at target during the school period, 39% and 67 respectively, compared to 84% and 98% in AHCL patients. AHCL systems can help reduce the metabolic control issues often detected during school hours. Our study suggests that, in the case of MDI use, the school period has a significant impact on the onset of hyperglycemic phenomena.

## 5. Conclusions

Our data confirm the annual trend of anti-correlation between temperatures and glycaemic trends and a general improvement in metabolic control in subjects who use AHCL microinfusion pumps throughout the year. The deterioration in metabolic control during heat waves is mitigated in subjects treated with AHCL microinfusion pumps. During the school period, subjects on MDI therapy show a greater tendency towards hyperglycaemia. During school days in particular, AHCL systems ensure better glycaemic levels. This is a useful goal in preventing long-term complications but also in improving the quality of pupils’ school life, considering the deleterious consequences of hyperglycaemia on cognitive abilities [47]. The reduction in severe hypoglycaemic episodes benefits the quality of school life for both families and school staff, who can also be supported by an extended system of caregivers thanks to the constant synchronisation of data, which is a useful resource, especially for younger children. It should be emphasised that fully exploiting opportunities for improving care requires specific educational strategies, and our work provides clear indications that educating families about MDI and AHCL should include information on blood glucose variations linked not only to seasonal temperature changes but also to acute disturbances caused by heat waves.

## Figures and Tables

**Table 1:** Demographical participant characteristics—clusters comparison.

**Number of Patients**	138	81
**Gender (M/F)**		
Males	47%	43%
Females	53%	57%
**Age**		
0–6 years	3%	0%
7–8 years	7%	2%
9–11 years	11%	9%
12–17 years	53%	19%
18–21 years	26%	7%
≥21 years	0%	63%

**Table 2:** Glycaemic variability was computed and, on this basis a second filtering was performed removing all patients characterised by a CV > 36%. % of patients characterised by CV target in table.

Season-Year	MDI	AHCL
Autumn-2022	33%	57%
Winter-2022	30%	59%
Spring-2023	30%	62%
Summer-2023	31%	58%

**Table 3:** Seasonal differences in glycaemic trends and frequency of hypoglycaemic and hyperglycaemic episodes. Variations in the CGM metrics in table.

Parameter	Recommended Value	% of Patients Autumn 2022 (MDI-AHCL)	% of Patients Winter 2022 (MDI-AHCL)	% of Patients Spring 2023 (MDI-AHCL)	% of Patients Summer 2023 (MDI-AHCL)
TAR (>250 mg/dL)	<5%	60–85%	55–83%	63–86%	60–83%
TAR (181–250 mg/dL)	<25%	78–96%	71–98%	73–92%	79–96%
TIR (70–180 mg/dL)	>70%	27–28%	26–42%	27–46%	21–45%
TBR (54–69 mg/dL)	<4%	100–98%	98–96%	98–98%	93–98%
TBR (<54 mg/dL)	<1%	100–96%	100–96%	100–96%	100–98%

**Table 4:** Statistical analysis of the differences between MDI and AHCL in the two periods (one year and heatwave 2023).

	One Year	Heatwave
Mean [mg/dL]	17.24	15.33
Std.Dev. [mg/dL]	6.04	19.18
RMSE [mg/dL]	18.26	24.55
Median [mg/dL]	17.13	19.72
Max [mg/dL]	34.04	45.96
MAD [mg/dL]	4.81	15.43

**Table 5:** Differences in glycaemic trends and frequency of hypoglycaemic and hyperglycaemic episodes between MDI and AHCL, for School Days (SD) and Not School Days (NSD). Variations in the CGM metrics in table.

Parameter	Recommended Value	% of Patients MDI-SD	% of Patients MDI-NSD	% of Patients AHCL-SD	% of Patients AHCL-NSD
CV	≤36%	39%	32%	62%	56%
TAR (>250 mg/dL)	<5%	67%	75%	84%	82%
TAR (181–250 mg/dL)	<25%	78%	93%	98%	98%
TIR (70–180 mg/dL)	>70%	19%	7%	34%	22%
TBR (54–69 mg/dL)	<4%	100%	98%	100%	98%
TBR (<54 mg/dL)	<1%	100%	100%	96%	98%

**Table 6:** Statistical analysis table on the differences between the two patterns in MDI (school days-not school days) and AHCL (school days-not school days).

	MDI	AHCL
Mean [mg/dL]	1.97	−1.14
Std.Dev. [mg/dL]	7.06	3.49
RMSE [mg/dL]	7.33	3.67
Median [mg/dL]	1.84	−1.34
Max [mg/dL]	17.94	10.51
MAD [mg/dL]	5.63	2.06

## Data Availability

Medical data are unavailable due to privacy and ethical restrictions. Meteorological data belong to “Centro Funzionale e Ufficio Idrologia, Idrografico, Mareografico—Agenzia di Protezione Civile della Regione Abruzzo”.
